# Reduced paucimannosidic *N*‐glycan formation by suppression of a specific β‐hexosaminidase from *Nicotiana benthamiana*

**DOI:** 10.1111/pbi.12602

**Published:** 2016-08-11

**Authors:** Yun‐Ji Shin, Alexandra Castilho, Martina Dicker, Flavio Sádio, Ulrike Vavra, Clemens Grünwald‐Gruber, Tae‐Ho Kwon, Friedrich Altmann, Herta Steinkellner, Richard Strasser

**Affiliations:** ^1^ Department of Applied Genetics and Cell BiologyUniversity of Natural Resources and Life SciencesViennaAustria; ^2^ Department of ChemistryUniversity of Natural Resources and Life SciencesViennaAustria; ^3^ NBM Inc.Wanju‐gunJeollabuk‐doKorea

**Keywords:** α1‐antitrypsin, glyco‐engineering, *N*‐glycosylation, *Nicotiana benthamiana*, plant‐made pharmaceuticals

## Abstract

Plants are attractive hosts for the production of recombinant glycoproteins for therapeutic use. Recent advances in glyco‐engineering facilitate the elimination of nonmammalian‐type glycosylation and introduction of missing pathways for customized *N*‐glycan formation. However, some therapeutically relevant recombinant glycoproteins exhibit unwanted truncated (paucimannosidic) *N*‐glycans that lack GlcNAc residues at the nonreducing terminal end. These paucimannosidic *N*‐glycans increase product heterogeneity and may affect the biological function of the recombinant drugs. Here, we identified two enzymes, β‐hexosaminidases (HEXOs) that account for the formation of paucimannosidic *N*‐glycans in *Nicotiana benthamiana*, a widely used expression host for recombinant proteins. Subcellular localization studies showed that HEXO1 is a vacuolar protein and HEXO3 is mainly located at the plasma membrane in *N. benthamiana* leaf epidermal cells. Both enzymes are functional and can complement the corresponding HEXO‐deficient *Arabidopsis thaliana* mutants. *In planta* expression of HEXO3 demonstrated that core α1,3‐fucose enhances the trimming of GlcNAc residues from the Fc domain of human IgG. Finally, using RNA interference, we show that suppression of HEXO3 expression can be applied to increase the amounts of complex *N*‐glycans on plant‐produced human α1‐antitrypsin.

## Introduction

The majority of therapeutic proteins including monoclonal antibodies, hormones and lysosomal enzymes are glycoproteins. For many glycoprotein drugs, a defined glycan structure is required for optimal efficacy. As a consequence, glycans from recombinant glycoproteins are considered as critical quality attributes by industry (Reusch and Tejada, 2015) and current manufacturing platforms are converted into systems with controllable glycosylation (Yang *et al*., 2015a). Recent progress in glyco‐engineering of plants has shown that *Nicotiana benthamiana* is highly suitable for the production of recombinant glycoproteins with tailor‐made *N*‐ and *O*‐glycan structures (Steinkellner and Castilho, 2015; Strasser *et al*., 2014). In particular, the glyco‐engineered ΔXT/FT mutant that exhibits stable down‐regulation of the plant enzymes β1,2‐xylosyl‐ and core α1,3‐fucosyltransferase has been used for the transient expression of different therapeutically relevant glycoproteins with custom‐made glycosylation (Castilho *et al*., 2014; Dicker *et al*., 2016; Jez *et al*., 2013; Loos *et al*., 2014, 2015; Schneider *et al*., 2014; Strasser *et al*., 2008, 2009; Wilbers *et al*., 2016). Notably, the ΔXT/FT plants are used to manufacture ZMapp the experimental antibody cocktail for treatment of acute Ebola virus infections (Qiu *et al*., 2014) and for the production of other monoclonal antibodies against infectious diseases that are currently under development (Loos *et al*., 2015; Zeitlin *et al*., 2016).

**Figure 1:**
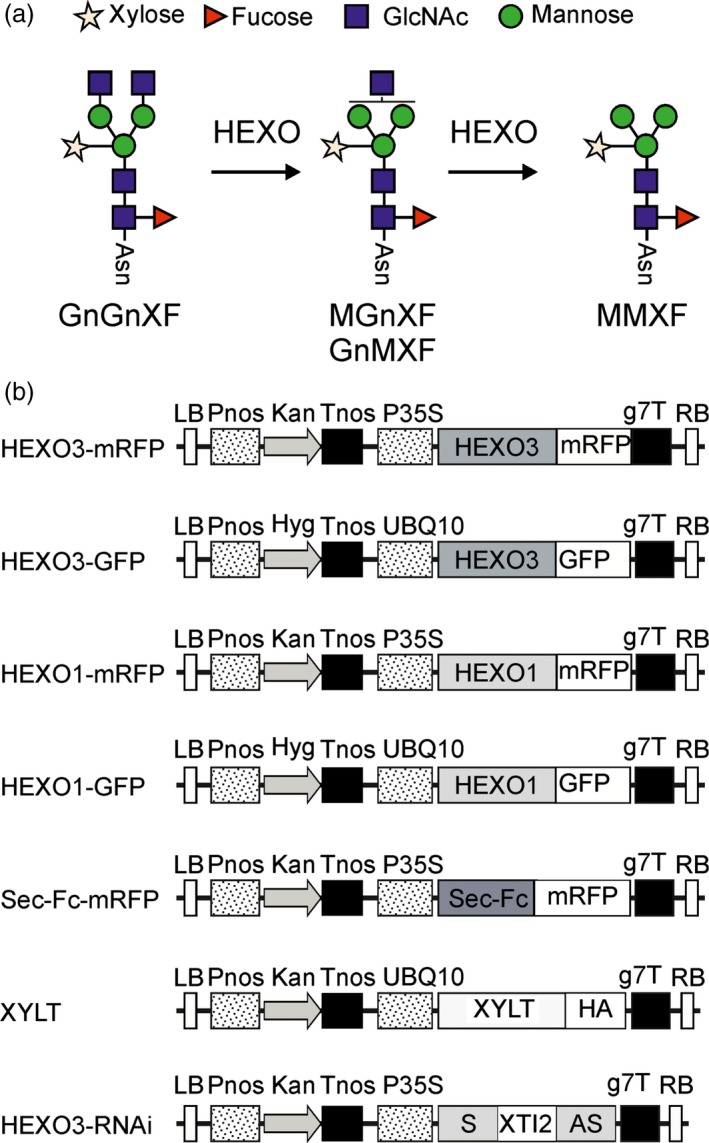
(a) Schematic illustration of proposed HEXO activity on complex *N*‐glycans. The symbols for the monosaccharides in the illustration are drawn according to the nomenclature from the Consortium for Functional Glycomics. (b) Schematic representation of used expression vectors. LB: left border; Pnos: nopaline synthase gene promoter; Hyg: hygromycin B phosphotransferase gene; Kan: neomycin phosphotransferase 2 gene; Tnos: nopaline synthase gene terminator; P35S: cauliflower mosaic virus 35S gene promoter; HEXO1: *Nicotiana benthamiana* HEXO1 open reading frame (ORF); HEXO3: *N. benthamiana* HEXO3 ORF; Sec‐Fc: signal peptide from α‐glucosidase II fused to the Fc domain of human IgG1; GFP: green fluorescent protein; mRFP: monomeric red fluorescent protein; XYLT:* Arabidopsis thaliana* β1,2‐xylosyltransferase ORF; HA: hemagglutinin tag; S: HEXO3 RNAi sequence in sense orientation; XTI2: intron 2 from *A. thaliana* XYLT; AS: HEXO3 RNAi sequence in antisense orientation; g7T: agrobacterium gene 7 terminator; RB: right border.

Complex *N*‐glycan formation is initiated in the *cis*/medial Golgi by *N*‐acetylglucosaminyltransferase I (GnTI) which transfers a single GlcNAc residue to Man_5_GlcNAc_2_ (Strasser *et al*., 1999). The presence of this terminal GlcNAc is a prerequisite for all further modifications including mannose trimming by Golgi‐α‐mannosidase II, transfer of a second terminal GlcNAc residue by *N*‐acetylglucosaminyltransferase II (GnTII), β1,2‐xylosylation and core α1,3‐fucosylation (Strasser, 2016). Commonly, these Golgi processing reactions result in the formation of complex *N*‐glycans with two terminal GlcNAc residues at the nonreducing end (GnGnXF: GlcNAc_2_XylFucMan_3_GlcNAc_2_ in wild‐type or GnGn: GlcNAc_2_Man_3_GlcNAc_2_ in ΔXT/FT plants) (Figure 1a). Such complex *N*‐glycans are, for example, the predominant structures on plant‐produced monoclonal antibodies. However, for other recombinant glycoproteins expression in *N. benthamiana* leaves resulted in the generation of *N*‐glycans with a considerable amount of truncated oligosaccharide structures (Castilho *et al*., 2014; Dicker *et al*., 2016; Dirnberger *et al*., 2001). These so‐called paucimannosidic *N*‐glycans (MMXF: Man_3_XylFucGlcNAc_2_ in wild‐type) expose terminal mannose residues at the nonreducing end. Under physiological conditions, paucimannosidic *N*‐glycans are quite rare on mammalian glycoproteins, but these truncated structures can be considerably increased in certain environments like cancer tissues (Schachter, 2009; Sethi *et al*., 2015). Moreover, exposed mannose residues on glycoproteins can accelerate their turnover by receptor‐mediated clearance from the blood (Yang *et al*., 2015b). For biotechnological production of most secreted recombinant glycoproteins, it is therefore relevant to prevent the formation of paucimannosidic *N*‐glycans.

As the majority of Golgi‐mediated *N*‐glycan processing steps are strictly dependent on the GlcNAc residue that is transferred by GnTI, the occurrence of paucimannosidic MMXF *N*‐glycans can only be explained by enzymatic removal of one or two terminal GlcNAc residues (Figure 1a). The site for this trimming reaction is most likely in a post‐Golgi compartment or in the apoplast. Plant β‐hexosaminidases (HEXOs) are the class of enzymes that can cleave off terminal GlcNAc residues from complex *N*‐glycans (Altmann *et al*., 1995). The *Arabidopsis thaliana* HEXO family consists of three members (AtHEXO1–AtHEXO3) (Liebminger *et al*., 2011; Strasser *et al*., 2007). AtHEXO1 is found mainly in the vacuoles where it generates paucimannosidic *N*‐glycans on vacuolar glycoproteins. AtHEXO2 activity and protein expression could not be detected in previous studies suggesting that AtHEXO2 represents an inactive or highly regulated enzyme that is expressed only in specific cell types. AtHEXO3, on the other hand, is an active β‐hexosaminidase located at the plasma membrane and acts on secreted glycoproteins (Castilho *et al*., 2014; Liebminger *et al*., 2011). Although these enzymes have been well characterized from *A. thaliana* and their ability to act on *N*‐glycans is well documented, their biological function is still unclear. Neither *hexo* single mutants, nor the *hexo1 hexo3* double mutant plants display any growth or developmental phenotype.

Expression of human α1‐antitrypsin (A1AT) in the *A. thaliana hexo3* mutant resulted in the formation of the fully processed GnGnXF *N*‐glycan instead of the paucimannosidic MMXF that was observed in wild‐type (Castilho *et al*., 2014). Consequently, HEXO3 activity is a major limitation for the production of distinct glycoproteins in plants as it generates unwanted truncated *N*‐glycans and contributes to the overall *N*‐glycan microheterogeneity. Here, we aimed to identify and inactivate the corresponding HEXOs from *N. benthamiana* plants. In addition, we performed *in planta* HEXO activity assays demonstrating that the attachment of core α1,3‐fucose can influence the processing of *N*‐glycans from a model glycoprotein. In summary, our study provides new insights into the specific function of plant HEXOs and paves the way for the efficient elimination of nonfavourable paucimannosidic *N*‐glycan formation in plants to improve the quality of plant‐made recombinant glycoproteins.

## Results

### A database search revealed several HEXO1 and HEXO3 candidates in *N. benthamiana*

The amino acid sequences from *A. thaliana* β‐hexosaminidases (AtHEXO1 and AtHEXO3) were used to search in the *N. benthamiana* draft genome for genes encoding putative HEXO orthologs. As the expression, enzymatic activity and putative function of AtHEXO2 is unclear (Strasser *et al*., 2007), no attempts were made to find orthologs from *N. benthamiana*. At least two different *N. benthamiana* sequences were identified which represent putative orthologs of AtHEXO1 and AtHEXO3. Based on this information, HEXO1 and HEXO3 coding regions were PCR amplified using cDNA derived from *N. benthamiana* leaf RNA. The obtained DNA sequence information suggested the amplification of a clone corresponding to a putative full‐length HEXO1 homolog from *N. benthamiana* that was also annotated in different *N. benthamiana* sequence databases (Figure S2). Despite several attempts, no sequence corresponding to an additional HEXO1 candidate was obtained. RT‐PCR from leaf RNA allowed the amplification of a complete open reading frame (ORF) corresponding to HEXO3. The cDNA sequence was slightly different from the annotation in the Sol Genomics Network database, but transcripts carrying the identified HEXO3 ORF were present in the *N. benthamiana* transcriptome database (Figure S3). RT‐PCR analyses for other HEXO3 candidates resulted in the amplification of several clones harbouring incomplete or aberrant cDNA fragments. We fully sequenced 12 different clones from two independent RT‐PCR amplification events, but we were unable to confirm the presence of an additional HEXO3 ORF. All the sequenced clones were interrupted by small deletions or insertions resulting in frame shifts and the generation of premature stop codons. As a consequence of these screening and cloning results, all further studies were performed with the clearly identified candidates for HEXO1 and HEXO3.

The HEXO1 ORF codes for a 541 amino acid protein with a predicted N‐terminal signal peptide sequence (amino acids 1–24). Seven potential *N*‐glycosylation sites are assigned on the protein backbone. The HEXO3 ORF encodes a 530 amino acid protein with a hydrophobic N‐terminal region that could either represent a signal peptide or a single transmembrane domain. The predictions for possible transmembrane domains are not consistent. While TMHMM, for example, does not predict a clear transmembrane domain, HMMTOP suggests the presence of an N‐terminal transmembrane helix and type II membrane protein topology with a large luminal catalytic domain. HEXO3 contains six possible *N*‐glycosylation sites whereby one carries a proline in +1 position (NPS site) and is therefore very likely not glycosylated. HEXO1 displays 71% identity to AtHEXO1 at the amino acid level and 51% identity to HEXO3. HEXO3 displays 71% identity to AtHEXO3.

### HEXO1 and HEXO3 are glycosylated proteins located in the vacuole and at the plasma membrane, respectively

**Figure 2:**
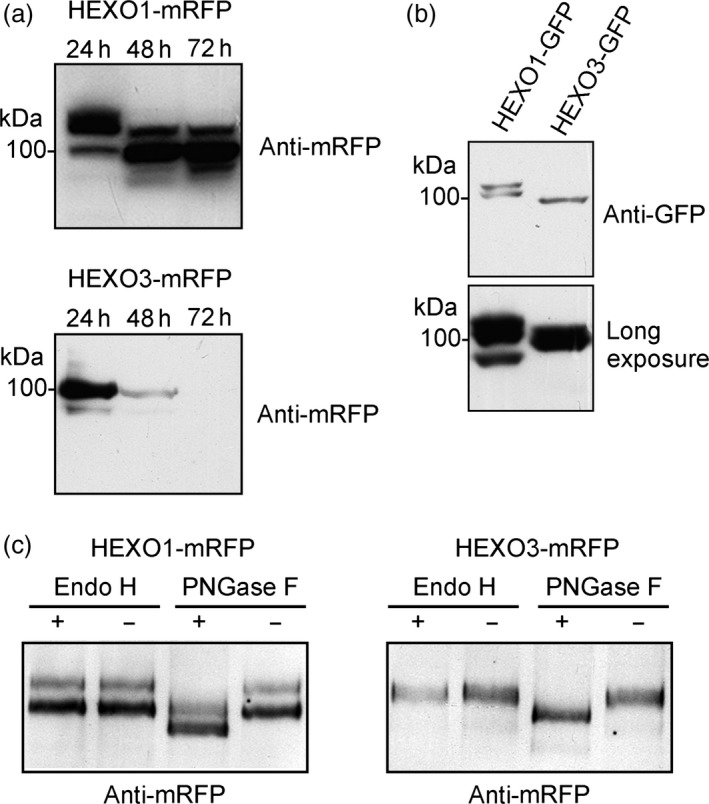
HEXO1 and HEXO3 are glycosylated with complex *N*‐glycans. Immunoblot analysis of transiently expressed HEXO variants: (a) HEXO1‐mRFP and HEXO3‐mRFP protein expression analysed at three different time points (24, 48 and 72 h postinfiltration). (b) HEXO1‐GFP and HEXO3‐GFP expression 48 h postinfiltration. (c) Endo H and PNGase F digestion of HEXO1‐mRFP and HEXO3‐mRFP.

To characterize the identified HEXO candidates, we cloned cDNA corresponding to the ORFs of HEXO1 and HEXO3 into plant expression vectors carrying the sequences for green and red fluorescent protein tags (Figure 1b). The constructs were transformed into agrobacterium and transiently expressed in *N. benthamiana* leaves by agroinfiltration. Leaf material was harvested 24, 48 and 72 h postinfiltration and analysed by immunoblotting (Figure 2a). HEXO1‐mRFP was clearly detectable at all three time points. However, the migration position of bands changed considerably from 24 to 48 h suggesting that HEXO1 is subjected to additional processing or post‐translational modifications. Likewise, immunoblot analysis of HEXO1‐GFP revealed the presence of two bands when samples were harvested 48 h after infiltration (Figure 2b). HEXO3‐mRFP was highly expressed at 24 h postinfiltration and showed like HEXO3‐GFP predominately a single band of the expected size of approximately 90–95 kDa (Figure 2a,b).

**Figure 3:**
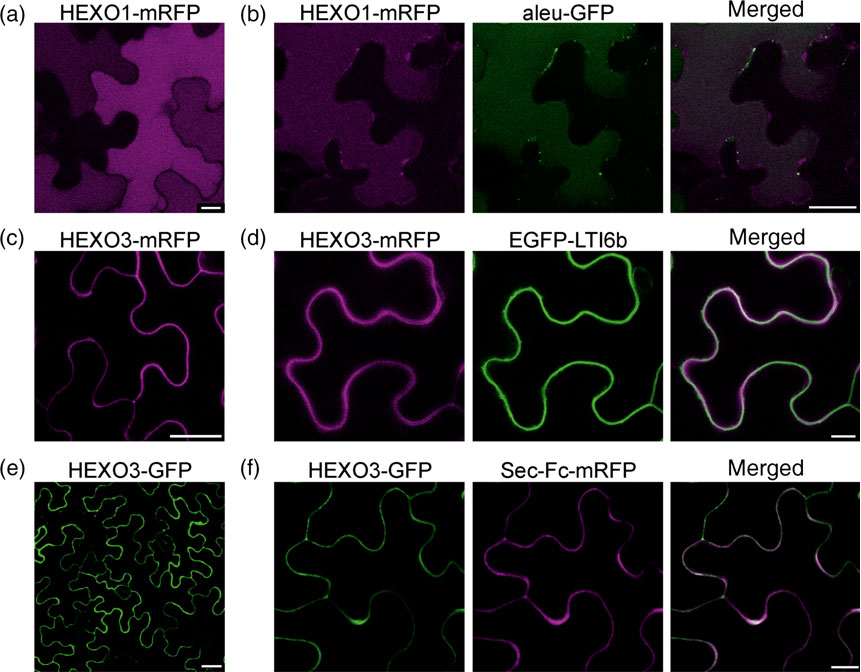
HEXO1 and HEXO3 are located in different subcellular compartments. Transient expression of HEXO1 and HEXO3 with or without different subcellular markers. (a) Confocal microscopy of HEXO1‐mRFP, scale bar = 10 μm. (b) Colocalization of HEXO1‐mRFP with aleu‐GFP, scale bar = 25 μm. (c) HEXO3‐mRFP, scale bar = 25 μm. (d) Colocalization of HEXO3‐mRFP with EGFP‐LTI6b, scale bar = 10 μm. (e) HEXO3‐GFP, scale bar = 25 μm. (f) Colocalization of HEXO3‐GFP with Sec‐Fc‐mRFP, scale bar = 10 μm.

To investigate the *N*‐glycosylation state of the expressed HEXO variants, Endo H and PNGase F digestions were carried out. Both proteins, HEXO1‐mRFP and HEXO3‐mRFP, were fully resistant to Endo H and sensitive to PNGase F when expressed in ΔXT/FT plants that generate negligible amounts of PNGase F‐resistant core α1,3‐fucosylated *N*‐glycans. These results show that HEXO1 and HEXO3 are glycosylated with Golgi‐processed *N*‐glycans (Figure 2c). The absence of Endo H‐sensitive ER‐derived oligomannosidic *N*‐glycans indicates efficient exit from the ER and trafficking trough the Golgi to their final destination. To examine the subcellular location of the two HEXO proteins, we analysed the expression of fluorescently tagged variants by confocal laser scanning microscopy. HEXO1‐mRFP displayed a clear vacuolar distribution (Figure 3a) that was also confirmed by colocalization with the vacuolar marker aleu‐GFP (Humair *et al*., 2001) (Figure 3b). These data suggest that HEXO1 resides in the large central vacuole in *N. benthamiana* leaf epidermal cells. By contrast, HEXO3‐mRFP labelled the outline of the epidermal cells indicating targeting to the plasma membrane and/or apoplast (Figure 3c). Co‐expression of HEXO3‐mRFP with the plasma membrane marker EGFP‐LTI6b (Kurup *et al*., 2005) (Figure 3d) or co‐expression of HEXO3‐GFP with the apoplast‐targeted glycoprotein Sec‐Fc‐mRFP (Figure 3e,f) revealed colocalization. In summary, the identified cellular sites are consistent with the ones shown for the corresponding AtHEXOs further suggesting that they are functional orthologs (Liebminger *et al*., 2011; Strasser *et al*., 2007). For many recombinant glycoproteins, secretion to the extracellular space is the preferred subcellular site for their accumulation in plants. Our subcellular localization studies suggest that HEXO3 is the candidate enzyme for trimming of terminal GlcNAc residues from secreted glycoproteins.

### HEXO1 and HEXO3 can complement *A. thaliana hexo* mutants

**Figure 4:**
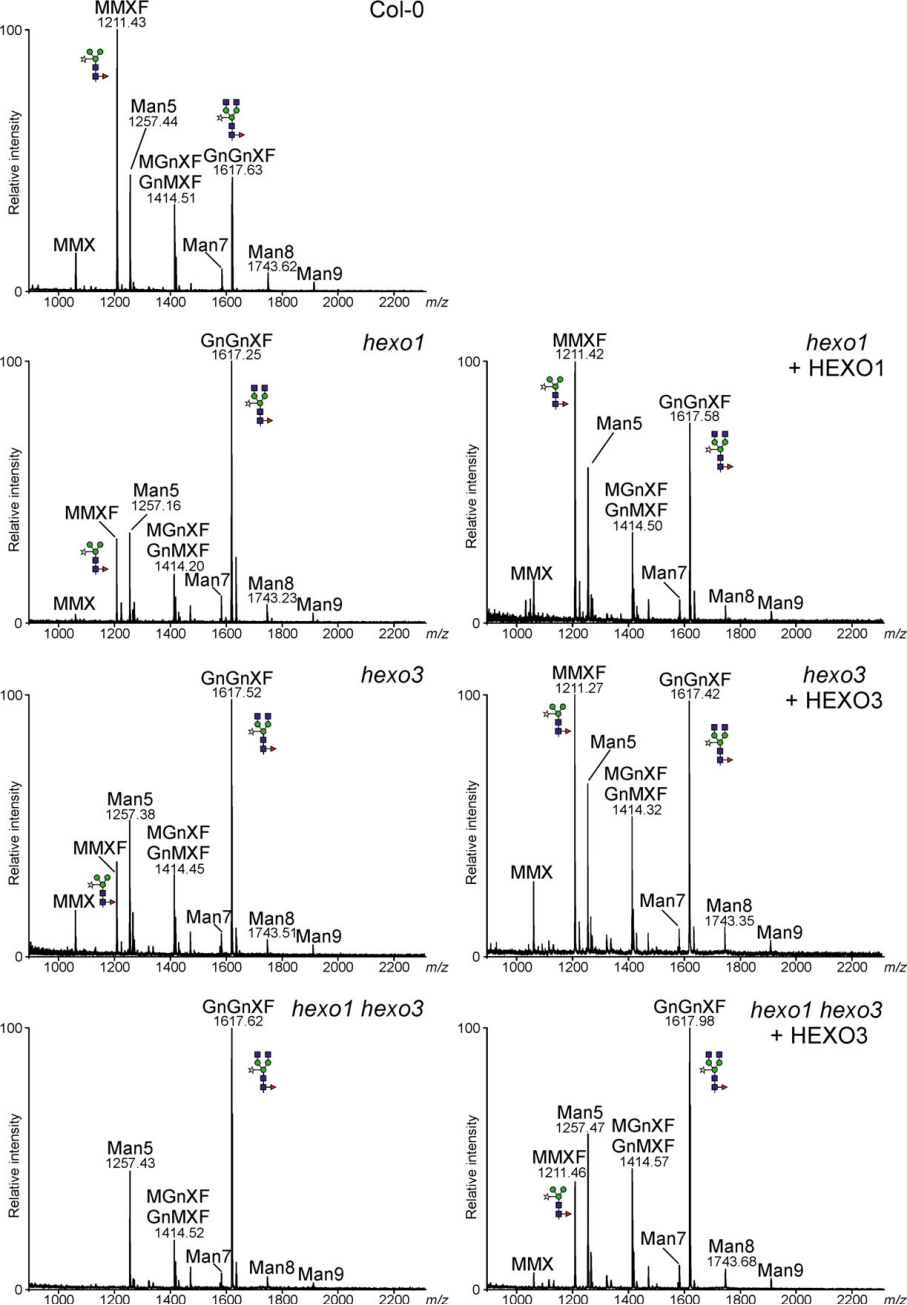
*Nicotiana benthamiana* HEXOs can complement the HEXO deficiency of *Arabidopsis thaliana hexo* mutants. Total *N*‐glycan analysis by MALDI‐MS of Col‐0 wild‐type, *hexo1*,* hexo1* expressing HEXO1‐GFP,* hexo3*,* hexo3* expressing HEXO3‐GFP,* hexo1 hexo3* and *hexo1 hexo3* expressing HEXO3‐GFP. Peaks were labelled according to the ProGlycAn system (www.proglycan.com).

To monitor functional activities of HEXOs, *A. thaliana* mutants that lack either endogenous AtHEXO1 (*hexo1*) or AtHEXO3 activities (*hexo3*) were used in complementation experiments (Liebminger *et al*., 2011). Transgenic *hexo1* and *hexo3* mutants that overexpress HEXO1‐GFP and HEXO3‐GFP, respectively, were generated, and total *N*‐glycans were analysed by MALDI mass spectrometry (Figure 4). Expression of HEXO1‐GFP resulted in a clear increase of paucimannosidic MMXF *N*‐glycans and a decrease of the complex *N*‐glycan GnGnXF. Similarly, HEXO3‐GFP could complement the HEXO deficiency of *hexo3* plants. The *A. thaliana hexo1 hexo3* double mutant lacks any detectable HEXO acting on *N*‐glycans and therefore does not produce paucimannosidic *N*‐glycans at all (Liebminger *et al*., 2011). To further confirm the enzymatic activity of HEXO3, complementation of *hexo1 hexo3* was analysed. Transgenic expression of HEXO3‐GFP resulted in the formation of substantial amounts of *N*‐glycans corresponding to the paucimannosidic MMXF structure in *hexo1 hexo3* that were not present in the double mutant (Figure 4). Collectively, the functional complementation and subcellular localization data demonstrate that HEXO1 and HEXO3 are functional orthologs of AtHEXO1 and AtHEXO3, respectively.

### Core α1,3‐fucose promotes HEXO3 activity

**Figure 5:**
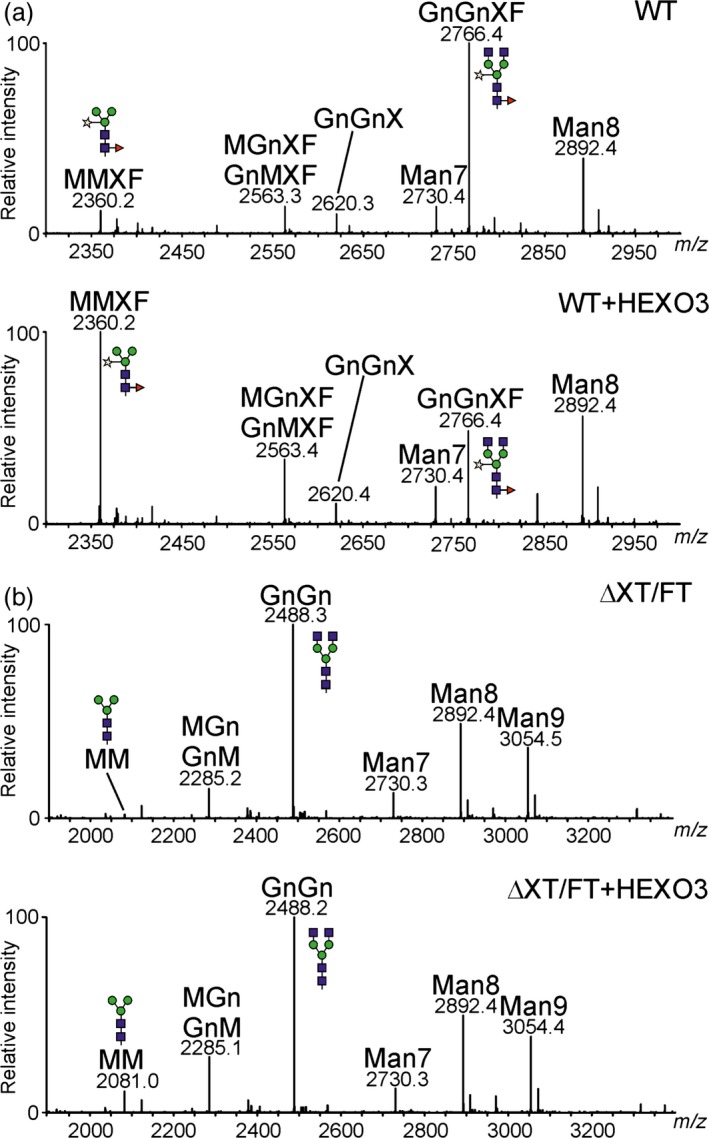
Overexpressed HEXO3 preferentially cleaves off GlcNAc residues from complex *N*‐glycans with β1,2‐xylose and core α1,3‐fucose. Transient co‐expression of HEXO3‐mRFP with Sec‐Fc‐mRFP (a) in WT or (b) in ΔXT/FT plants. Sec‐Fc‐mRFP was purified, digested with trypsin and the glycosylated peptide EEQYNSTYR was analysed by LC‐ESI‐MS.

The subcellular localization analysis and data from complementation experiments indicate that HEXO3 is responsible for the cleavage of GlcNAc residues in the apoplast or during the trafficking of proteins from the Golgi to the extracellular space. To obtain more evidence that HEXO3 can act on recombinant glycoproteins, we transiently co‐expressed HEXO3‐mRFP with a glycoprotein in *N. benthamiana* leaves and analysed whether the overexpression increases the GlcNAc trimming from *N*‐glycans. First, we have chosen a model glycoprotein (Sec‐Fc‐mRFP) containing a signal peptide, the Fc part from human IgG1, carrying a single *N*‐glycosylation site, and mRFP (Figure 1b). As confirmed by confocal laser scanning microscopy, Sec‐Fc‐mRFP is targeted to the secretory pathway and accumulates in the apoplast (Figure 3f). When expressed in *N. benthamiana* wild‐type, predominately GnGnXF structures were detected on the purified protein (Figure 5a). Likewise, when expressed in ΔXT/FT, the Fc *N*‐glycosylation site carried mainly GnGn and virtually no paucimannosidic or incompletely processed complex *N*‐glycans (Figure 5b). Co‐expression of Sec‐Fc‐mRFP and HEXO3‐mRFP in ΔXT/FT did not significantly alter the overall *N*‐glycan profile. However, in *N. benthamiana* wild‐type, the co‐expression of HEXO3‐mRFP resulted in a marked increase of paucimannosidic MMXF structures showing that HEXO3‐mRFP is active when transiently co‐expressed with a glycoprotein (Figure 5a).

**Figure 6:**
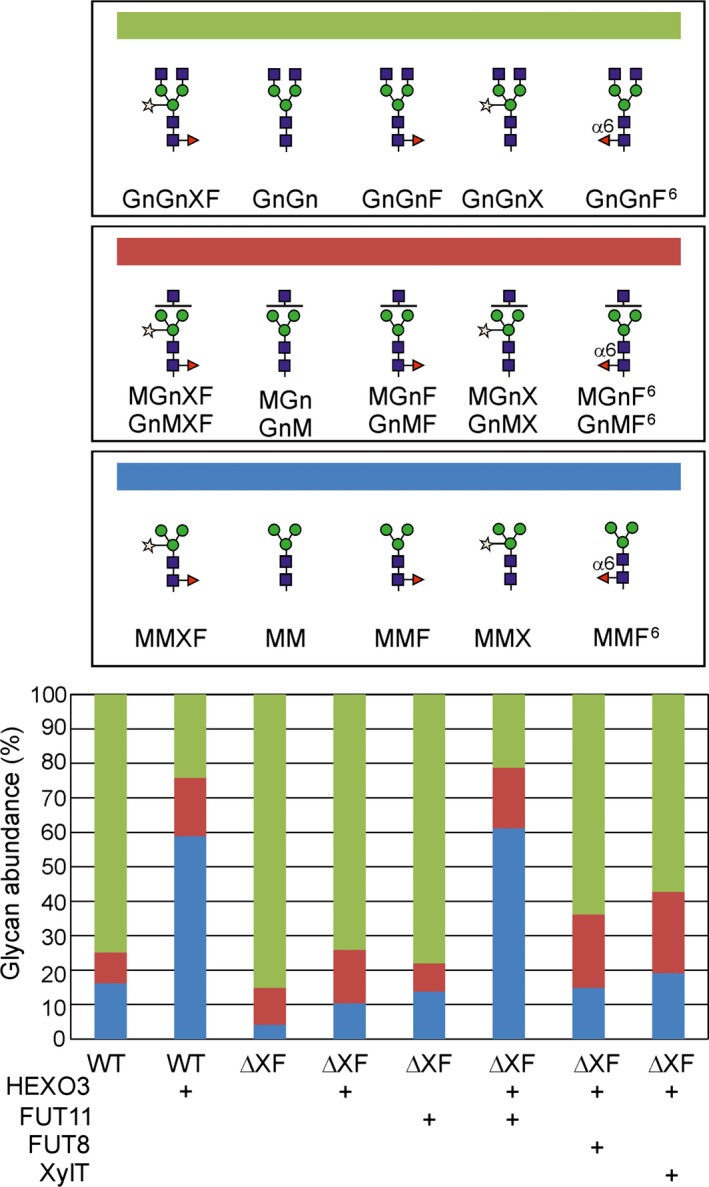
The presence of core α1,3‐fucose enhances the trimming of GlcNAc from the Fc *N*‐glycan. Relative abundance of complex and paucimannosidic *N*‐glycans upon co‐expression of Sec‐Fc‐mRFP, HEXO3‐mRFP and different *N*‐glycan processing enzymes (FUT11: *Arabidopsis thaliana* core α1,3‐fucosyltransferase; FUT8: mouse core α1,6‐fucosyltransferase; XylT: *A. thaliana* β1,2‐xylosyltransferase). Tryptic digested glycopeptides from the Fc domain (EEQYNSTYR) were analysed by LC‐ESI‐MS. Different shades of blue, red and green represent percentages of paucimannosidic structures, complex *N*‐glycans with one terminal GlcNAc residue and complex *N*‐glycans with two terminal GlcNAc residues, respectively. Mean values from two to three biological replicates are shown. The corresponding *N*‐glycan structures are indicated.

The observed difference between wild‐type and ΔXT/FT plants was unexpected and suggested that the presence of either β1,2‐xylose or core α1,3‐fucose promote trimming by HEXO3. To test this assumption, we transiently co‐expressed Sec‐Fc‐mRFP and HEXO3‐mRFP with the *A. thaliana* β1,2‐xylosyltransferase (XYLT), *A. thaliana* core α1,3‐fucosyltransferase (FUT11) or mouse core α1,6‐fucosyltransferase (FUT8) and analysed the effect on GlcNAc removal by LC‐ESI‐MS. While neither the presence of β1,2‐xylose nor the presence of core α1,6‐fucose caused an increase in terminal GlcNAc processing from the Fc *N*‐glycosylation site, the presence of core α1,3‐fucose resulted in the formation of considerable amounts of paucimannosidic *N*‐glycans (Figure 6,S4). Consequently, our data show that the presence of an additional sugar in a specific linkage (core α1,3‐fucose) causes alterations in *N*‐glycan processing by HEXO3.

### Knock‐down of HEXO3 leads to increased amounts of complex *N*‐glycans on recombinant A1AT

**Figure 7:**
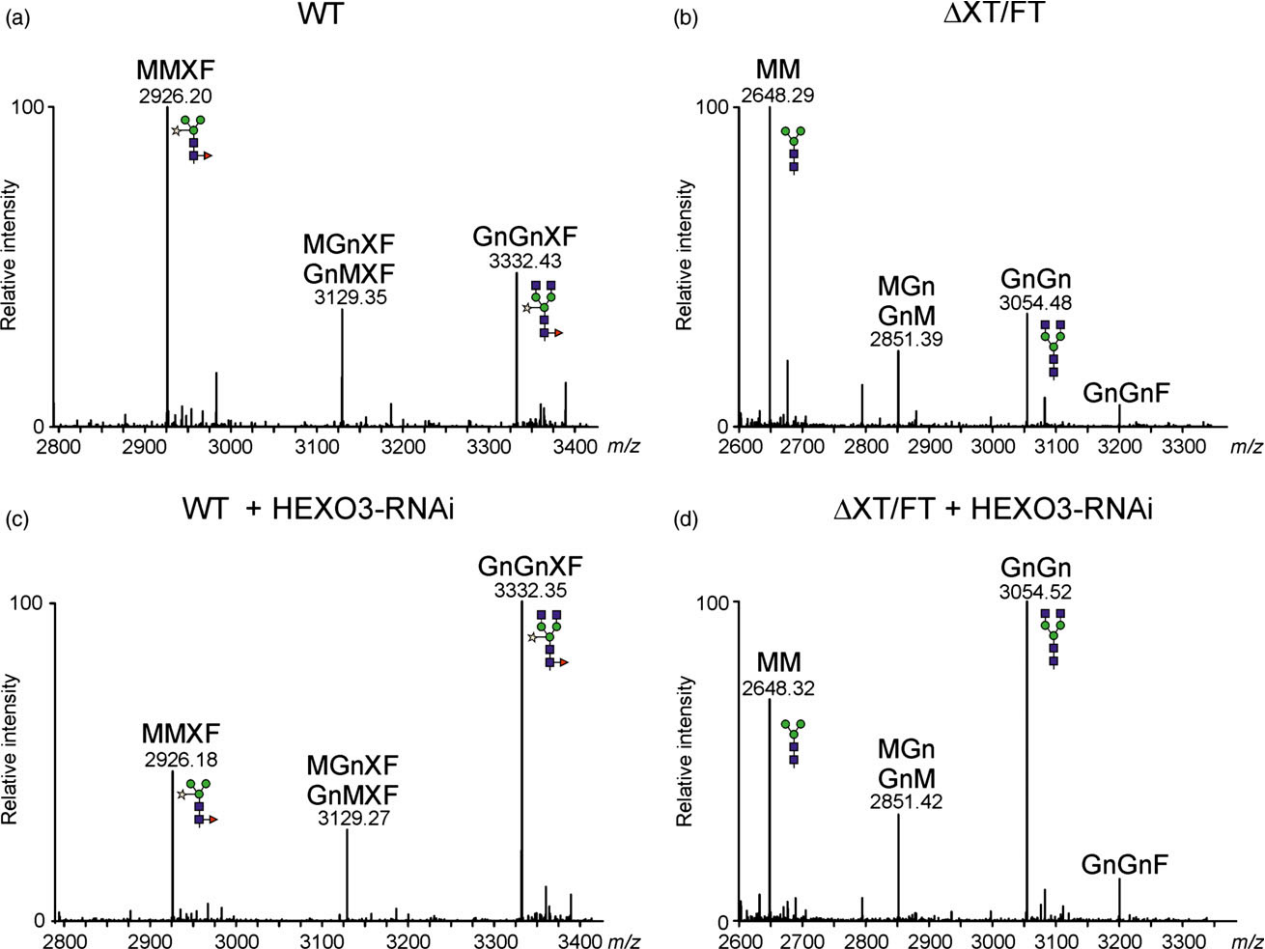
Transient co‐expression of the HEXO3‐RNAi construct leads to enhanced complex *N*‐glycan formation on secreted A1AT. Human A1AT was transiently expressed in (a, c) WT or (b, d) ΔXT/FT plants, in the absence (a, b) or presence (c, d) of the HEXO3 silencing construct. LC‐ESI‐MS of trypsin‐digested A1AT collected from the IF 3 days postinfiltration. The *N*‐glycosylation profile of glycopeptide 3 (^243^ YLGNATAIFFLPDEGK ^259^) is shown.

Based on the subcellular localization and data from *in planta* activity of co‐expressed HEXO3, we hypothesized that reduction or complete elimination of HEXO3 activity will reduce the amounts of paucimannosidic *N*‐glycans on secreted recombinant glycoproteins. To investigate whether the formation of paucimannosidic *N*‐glycans can be blocked, we designed an RNAi construct for silencing of HEXO3. The HEXO3‐RNAi construct was infiltrated into *N. benthamiana* wild‐type and ΔXT/FT leaves. *N*‐glycans from leaf extracts and isolated intercellular fluid (IF) were analysed 3 days postinfiltration by mass spectrometry. The complex *N*‐glycans from total soluble proteins of wild‐type and ΔXT/FT were increased in the presence of HEXO3‐RNAi (Figure S5). Likewise, the transient expression of HEXO3‐RNAi resulted in a decrease of paucimannosidic *N*‐glycans on IF‐derived proteins (Figure S6). To test the approach on a therapeutically interesting recombinant glycoprotein, we co‐expressed human A1AT (Castilho *et al*., 2014) together with the HEXO3‐RNAi construct. *N*‐glycans of recombinant A1AT extracted from the IF were analysed by LC‐ESI‐MS. In the absence of HEXO3‐RNAi, considerable amounts of paucimannosidic MMXF and MM structures were found in wild‐type (Figure 7a) or ΔXT/FT plants (Figure 7b and Table 1). Co‐expression of HEXO3‐RNAi led to a profound increase of GnGnXF (Figure 7c) and GnGn *N*‐glycans (Figure 7d,S7). In summary, these results demonstrate that *N. benthamiana* HEXO3 activity is a critical factor that generates truncated *N*‐glycans on secreted recombinant glycoproteins in plants.

**Table 1:** Relative amounts (%) of paucimannosidic (MM, MMXF), complex with one (GnM/MGn, GnMXF/MGnXF) or complex with two (GnGn, GnGnXF) GlcNAc residues on glycopeptide 2 (GP2) or 3 (GP3) from recombinant A1AT

Glycan	GP2	GP3	GP2	GP3
	ΔXTFT^a^		ΔXTFT + HEXO3 RNAi^a^	
MM	57.9	53.3	36.6	34.5
GnM/MGn	16.2	15.9	16.7	14.4
GnGn	25.9	30.8	46.7	51.0

	WT^b^		WT + HEXO3 RNAi^b^	
MMXF	39.8	45.7	37.3	26.3
GnMXF/MGnXF	27.7	17.3	21.9	16.6
GnGnXF	32.5	37	40.8	57.1

a Mean values from two independent biological replicates are shown.

b Amounts for WT are derived from a single analysis.

## Discussion

*N. benthamiana* plants are a key expression platform for the production of recombinant proteins (Qiu *et al*., 2014; Stoger *et al*., 2014; Strasser *et al*., 2014). In this study, we identified and characterized HEXOs that trim terminal GlcNAc residues from vacuolar or secreted glycoproteins in *N. benthamiana*. For the production of distinct recombinant glycoproteins, active HEXOs are a severe limitation because these enzymes generate truncated *N*‐glycans that are not common on mammalian glycoproteins. Moreover, as HEXO enzymes are trafficking through the Golgi on their journey to their final destination and display β‐hexosaminidase activity in the pH‐milieu of the Golgi (Strasser *et al*., 2007), it is possible that HEXOs cleave GlcNAc residues already in one of the Golgi subcompartments. Notably, insect cells that generate similar paucimannosidic *N*‐glycans have a processing β‐hexosaminidase that is found in the Golgi apparatus (Altmann *et al*., 1995; Léonard *et al*., 2006). GlcNAc removal in the Golgi interferes with other *N*‐glycan processing reactions leading to increased *N*‐glycan heterogeneity and may prevent further elongations with β1,4‐galactose or sialic acid.

Our data confirm previous results indicating the existence of an active HEXO in the apoplast or plasma membrane that acts in a protein‐specific manner (Castilho *et al*., 2014; Liebminger *et al*., 2011; Strasser *et al*., 2007). Several recombinant human glycoproteins have been expressed in *N. benthamiana* leaves, and it seems that only some glycoproteins are substrates for HEXOs suggesting that the glycoprotein conformation or the interaction between the protein backbone and the attached glycan prevent processing. Due to the special structural features of the Fc homodimer, the single Fc *N*‐glycan of human IgG1 appears, for example, quite resistant to processing by HEXOs. The same has been described for human EPO‐Fc and transferrin when transiently expressed in *N. benthamiana* (Castilho *et al*., 2011). These proteins carry mainly fully processed GlcNAc terminating complex *N*‐glycans. By contrast, human A1AT harbours considerably amounts of paucimannosidic structures on all three *N*‐glycosylation sites (Castilho *et al*., 2014). A similar result was observed for the *N*‐glycans of recombinantly expressed follicle stimulating hormone, recombinant glucocerebrosidase targeted to the apoplast or human lactoferrin (Dirnberger *et al*., 2001; He *et al*., 2012; Limkul *et al*., 2016; Samyn‐Petit *et al*., 2003). Further studies are needed to identify the protein intrinsic features that lead to efficient processing by plant HEXOs.

Interestingly, the impact of core α1,3‐fucose on GlcNAc trimming shows that specific *N*‐glycans on proteins like the Fc domain‐containing Sec‐Fc‐mRFP can be converted into HEXO3 substrates. The impact of core α1,3‐fucose on *N*‐glycan modifications has been described recently for the *N*‐glycan from the Fc domain of the monoclonal antibody cetuximab (Castilho *et al*., 2015). In addition to the conserved Fc *N*‐glycan, cetuximab has a second *N*‐glycan in the variable domain of the heavy chain. These two *N*‐glycosylation sites on the heavy chain allow the comparison of *N*‐glycan processing in a given cell in the presence or absence of an additional glycan modification. As a result of this study, it was shown that core α1,3‐fucosylation increases branching, bisecting GlcNAc formation and in particular sialylation of the Fc *N*‐glycan presumably by alleviating structural constraints between the Fc *N*‐glycan and the IgG1 CH2 domains (Castilho *et al*., 2015).

Co‐expression of the HEXO3‐RNAi construct resulted in a reduction of paucimannosidic structures, while complex *N*‐glycans increased. However, some paucimannosidic *N*‐glycans were still detectable. One reason could be that the used transient silencing approach is not very efficient. It is plausible that residual HEXO3 protein is present in the apoplast that has been made before the HEXO3 silencing was established. An optimization of the infiltration procedure like pre‐infiltration with the RNAi construct at an earlier time point and subsequent infiltration with the recombinant glycoprotein could significantly improve the down‐regulation of the unwanted HEXO activity. Moreover, the production of stable HEXO3 silencing lines is ongoing and genome editing will be applied to completely inactivate HEXO3 in *N. benthamiana* in the future. The complete HEXO3 knockout will finally show whether the identified HEXO3 candidate is the only one acting on complex *N*‐glycans or whether other HEXO candidates are also functional and contribute to the formation of paucimannosidic *N*‐glycans on secreted glycoproteins.

For some therapeutic applications, it is beneficial to produce *N*‐glycans with increased amounts of paucimannosidic *N*‐glycans. This is, for example, the case for the carrot cell‐based production of the recombinant glucocerebrosidase taliglucerase alfa that is used for treatment of Gaucher's disease (Shaaltiel *et al*., 2007). The major *N*‐glycan structures found on taliglucerase alfa are paucimannosidic MMXF with β1,2‐xylose and core α1,3‐fucose (Tekoah *et al*., 2013). The exposed terminal mannose residues from recombinant glucocerebrosidase are essential for the internalization of the enzyme by macrophages. The *N*‐glycans on mammalian cell‐derived recombinant lysosomal enzymes have to be remodelled postproduction with glycosidases to obtain exposed mannose residues (Grabowski *et al*., 1995). By contrast, for taliglucerase alfa, the efficient production of *N*‐glycans with terminal mannose was achieved by targeting to the vacuole using a seven amino acid long vacuolar targeting signal fused to the recombinant glucocerebrosidase (Shaaltiel *et al*., 2007). Using HEXO3 overexpression, the attachment of non‐native targeting signals can be avoided and exposed mannose residues can be generated on secreted recombinant glycoproteins (Shen *et al*., 2016).

In conclusion, we have identified active HEXOs from *N. benthamiana* and found that HEXO3 contributes to the formation of paucimannosidic *N*‐glycans on secreted recombinant glycoproteins. Furthermore, we provide novel glyco‐engineering tools that can be applied to eliminate paucimannosidic *N*‐glycan formation or alternatively, generate mannose‐terminated *N*‐glycans on secreted glycoproteins. Implementation of these tools into the existing *N. benthamiana* expression platforms will increase the capacity of this plant for the expression of therapeutic glycoproteins for many different applications.

## Experimental procedures

#### Cloning of N. benthamiana HEXO candidates

The *A. thaliana* HEXO1 (At3g55260) and HEXO3 (At1g65590) amino acid sequences were used to search in different *N. benthamiana* genome databases using tBLASTN (https://solgenomics.net/organism/Nicotiana_benthamiana/genome; http://benthgenome.qut.edu.au/). Based on the retrieved sequences, primers were designed and used for RT‐PCR to amplify full‐length ORFs coding for HEXO proteins. For this purpose, RNA was extracted from leaves of 4‐ to 5‐week‐old *N. benthamiana* using the SV Total RNA Isolation System (Promega, Mannheim Germany) and the iScript cDNA Synthesis Kit (Bio‐Rad, Vienna Austria). An aliquot of the cDNA was used for the PCR amplification with Phusion High‐Fidelity DNA Polymerase (Biozym, Hessisch Oldendorf, Germany) and different primer combinations (Table S1). PCR products were ligated into a cloning vector using the Zero Blunt Topo PCR Cloning Kit (Thermo Fisher Scientific, Vienna, Austria) and fully sequenced. Sequence alignments were made using SeqMan II software (DNASTAR Lasergene, Madison, WI, USA).

#### Transient expression and immunoblots

For expression of *N. benthamiana* HEXO1 in plants, the corresponding ORF was amplified by PCR from the cloning vector using primers Nb‐Hexo1‐F4 and Nb‐Hexo1‐R4. The PCR product was *Spe*I/*Bam*HI digested and cloned into *Xba*I/*Bam*HI digested plant expression vectors p31 (Schoberer *et al*., 2013) and p47 (Hüttner *et al*., 2014) to generate p31‐NbHEXO1 and p47‐NbHEXO1. In p31 expression is under the control of the CaMV 35S promoter and the recombinant protein is C‐terminally fused to mRFP, whereas in p47 expression is under the control of the *A. thaliana* ubiquitin 10 promoter and the recombinant protein is C‐terminally fused to GFP (Figure 1b). For expression of HEXO3, the corresponding ORF was amplified by PCR from the cloning vector with Nb‐Hexo3‐F6 and Nb‐Hexo3‐R5. The PCR product was *Xba*I/*Bam*HI digested and cloned into *Xba*I/*Bam*HI digested vectors p31 and p47 to generate p31‐NbHEXO3 and p47‐NbHEXO3. The plant expression vectors were transformed into *Agrobacterium tumefaciens* (strain UIA143 was used for all constructs). Syringe‐mediated agroinfiltration was used for transient expression in leaves of 4‐ to 5‐week‐old *N. benthamiana* plants. At the indicated time points, leaf pieces were harvested from infiltrated plants and total protein extracts were prepared by grinding of frozen leaves with a mixer mill and steel balls. The ground leaves were dissolved in RIPA buffer (Sigma‐Aldrich, Vienna, Austria) followed by centrifugation at 16 000 ***g*** for 10 min. An aliquot of the supernatant was mixed with SDS‐PAGE loading buffer, denatured at 95 °C for 5 min and subjected to SDS‐PAGE under reducing conditions. Protein gel blots were blocked in PBS containing 0.1% (v/v) Tween 20 and 3% (w/v) BSA. The membranes were probed with anti‐GFP‐horseradish peroxidase (MACS Miltenyi Biotec, Bergisch Gladbach, Germany) or anti‐mRFP (Chromotek, Planegg‐Martinsried, Germany) antibodies. Endo H (New England Biolabs, Frankfurt am Main, Germany) and PNGase F (New England Biolabs) digestions were performed as described in detail recently (Hüttner *et al*., 2014).

#### Confocal imaging of fluorescent protein fusions

Leaves of 4‐ to 5‐week‐old *N. benthamiana* plants were infiltrated with agrobacterium suspensions carrying binary plant expression vectors for GFP‐ or mRFP‐tagged proteins with the following optical densities (OD600): 0.1 for p31‐NbHEXO1 (HEXO1‐mRFP), p31‐NbHEXO3 (HEXO3‐mRFP) and p47‐HEXO3 (HEXO3‐GFP). Constructs for aleu‐GFP (infiltrated with OD600 = 0.01) and for EGFP‐LTI6b (infiltrated with OD600 = 0.1) were available from a previous study (Strasser *et al*., 2007). The p39‐Sec‐Fc‐mRFP construct was generated as follows: the DNA coding for the Fc domain from human IgG1 was amplified from p20F‐Fc (Schoberer *et al*., 2009) with primers Fc‐1F/Fc‐2R, *Bam*HI/*Bgl*II digested and cloned into the *Bam*HI site of p31‐Sec‐mRFP. To generate the vector p31‐Sec‐mRFP, a DNA fragment derived from annealing of primers GCSII_SP_F and GCSII_SP_R was ligated into the *Xba*I/*Bam*HI sites of vector p31. The fragment derived from these primers encodes the signal peptide of *A. thaliana* α‐glucosidase II (GCSII).

#### Complementation of A. thaliana hexo mutants

*Arabidopsis thaliana hexo* single and *hexo1 hexo3* double knockout plants (Liebminger *et al*., 2011) were transformed with p47‐NbHEXO1 or p47‐NbHEXO3 by floral dipping, as described previously (Strasser *et al*., 2004). Hygromycin‐resistant plants were screened by PCR with HEXO1‐ and HEXO3‐specific primers, respectively. Leaves from different PCR‐positive plants were pooled, and 500 mg was used for total *N*‐glycan analysis. Preparation of *N*‐linked glycans and matrix‐assisted laser desorption ionization (MALDI) mass spectrometry was carried out as described previously (Strasser *et al*., 2004).

#### In planta N‐glycan processing

The p39‐Sec‐Fc‐mRFP vector expressing the glycoprotein reporter was either expressed alone in *N. benthamiana* leaves by agroinfiltration or in combination with p31‐NbHEXO3 and additional constructs for expression of different glycosyltransferases. Vectors for plant expression of *A. thaliana* core α1,3‐fucosyltransferase 11 (FUT11) and mouse core α1,6‐fucosyltransferase (FUT8) were available from a previous study (Castilho *et al*., 2015). The *A. thaliana* β1,2‐xylosyltransferase (XYLT) was amplified from *A. thaliana* cDNA using primers ARA_XT27F and ARA_XT29R. The PCR product was digested with *Spe*I/*Bam*HI and cloned into the *Xba*I/*Bam*HI site of p41 (a derivative of pPT2 with the UBQ10 promoter instead of the CaMV 35S promoter and a C‐terminal HA tag for monitoring of protein expression) to generate p41‐XYLT (Figure 1b). Two days postinfiltration 500 mg leaves were harvested, frozen leaves were grinded using a mixer mill and proteins were extracted with RIPA buffer. The Fc domain glycoprotein reporter was purified from the extract by binding to rProtein A Sepharose^™^ Fast Flow (GE Healthcare Europe, Vienna, Austria) as described in detail recently (Schoberer *et al*., 2014). Purified protein was subjected to SDS‐PAGE and Coomassie blue staining. The corresponding protein band was excised from the gel, destained, carbamidomethylated, in‐gel trypsin digested and analysed by liquid chromatography electrospray ionization mass spectrometry (LC‐ESI‐MS), as described in detail previously (Stadlmann *et al*., 2008). A detailed explanation of *N*‐glycan abbreviations can be found at http://www.proglycan.com.

#### Transient knockdown of HEXO3

A synthetic DNA fragment consisting of intron 2 from *A. thaliana* XYLT (Strasser *et al*., 2004, 2008) and an antisense DNA fragment corresponding to the coding sequence for amino acids 136–208 of HEXO3 was obtained by GeneArt gene synthesis (Thermo Fisher Scientific) (Figure S1). The obtained vector with the synthetic DNA was used as a template for PCR with primers Nb‐HEXO3‐F8 and Nb‐HEXO3‐R8. The ‘sense’ PCR product was digested with *Xba*I/*Kpn*I and cloned into the synthetic DNA containing vector to generate a sense–intron–antisense hairpin construct. The sense–intron–antisense sequence was subsequently excised by *Xba*I/*Bam*HI digestion and ligated into *Xba*I/*Bam*HI digested plant expression vector pPT2 (Strasser *et al*., 2007). In this vector, the RNAi construct is expressed under the control of the CaMV 35S promoter. The pPT2‐NbHEXO3‐RNAi vector was transformed into agrobacteria and transiently expressed by agroinfiltration in *N. benthamiana* leaves. Total *N*‐glycan analysis was performed 3 days after infiltration as described for *A. thaliana*. Expression of human A1AT, extraction from the IF and LC‐ESI‐MS analysis of glycopeptides was performed as described in detail recently (Castilho *et al*., 2014).

## Conflict of interest

The authors declared that they have no conflict of interests.

## Supporting information


**Figure S1** Synthetic DNA for cloning of the HEXO3 RNAi construct. The used restriction sites (XbaI, KpnI and BamHI) are highlighted. The sequence of the intron derived from A. thaliana β1,2‐xylosyltransferase is shown in red. The design of the construct is based on Strasser *et al*., 2008.
**Figure S2** Amino acid sequence alignment of HEXO1 sequences.
**Figure S3** Amino acid sequence alignment of HEXO3 sequences.
**Figure S4** The presence of core α1,3‐fucose enhances the trimming of GlcNAc from the Fc *N*‐glycan.
**Figure S5** Effect of HEXO3‐RNAi on *N*‐glycans from total soluble proteins.
**Figure S6** Effect of HEXO3‐RNAi on *N*‐glycans from glycoproteins of the intercellular fluid (IF).
**Figure S7** Transient co‐expression of human A1AT with HEXO3‐RNAi. LC‐ESI‐MS analysis of glycopeptide 2 from human A1AT.
**Table S1** List of all used primers in this study.
