# Supplementary material for: Reduced paucimannosidic N‐glycan formation by suppression of a specific β‐hexosaminidase from Nicotiana benthamiana
Source: Plant Biotechnol J. 2016 Aug 11;15(2):197–206. doi: 10.1111/pbi.12602 (PMC5259580; doi:10.1111/pbi.12602)
Supplement: Supplementary file 1 — Figure S1 Synthetic DNA for cloning of the HEXO3 RNAi construct. The used restriction sites (XbaI, KpnI and BamHI) are highlighted. The sequence of the intron derived from A. thaliana β1,2‐xylosyltransferase is shown in red. The design of the construct is based on Strasser et al., 2008. Figure S2 Amino acid sequence alignment of HEXO1 sequences. Figure S3 Amino acid sequence alignment of HEXO3 sequences. Figure S4 The presence of core α1,3‐fucose enhances the trimming of GlcNAc from the Fc N‐glycan. Figure S5 Effect of HEXO3‐RNAi on N‐glycans from total soluble proteins. Figure S6 Effect of HEXO3‐RNAi on N‐glycans from glycoproteins of the intercellular fluid (IF). Figure S7 Transient co‐expression of human A1AT with HEXO3‐RNAi. LC‐ESI‐MS analysis of glycopeptide 2 from human A1AT. Table S1 List of all used primers in this study. [file PBI-15-197-s001.pdf]

```
TTTTCTAGATATATAGGTACCACACTGCACGGTATGCTCCTCTTCTTGTTCATGGTCATGATCCTTATATGAGCAGGGAA
AGTCCAGTTTAGACTTGTAGTTAGTTACTCTTCGTTATAGGATTTGGATTTCTTGCGTGTTTATGGTTTTAGTTTCCCTC
CTTTGATGAATAAAATTGAATCTTGTATGAGTTTCATATCCATGTTGTGAATCTTTTTGCAGACGCAGCTAGTCAGTTTT
GCATAAGCCATTGAATCGATAACCTTCAATATCACAGGCAACGGCAGATAGTGACGGGAAGTATCAATTAAAAGCCCTCG
ATAAGAGAATCTTGGTCGATCAACTATGGTCCATGGAACTTGATGAACTTCAATTGTTCTGGTTGTAAAGTTAAAATGGC
ATACTTGACTAAATGTCTGCAAACCATGCAACGCCCCATAAACTGTTTTTGCGGATCC
```

Figure S1. Synthetic DNA for cloning of the HEXO3 RNAi construct. The used restriction sites (*Xba*I, *Kpn*I and *Bam*HI) are highlighted. The sequence of the intron derived from *A. thaliana* β1,2-xylosyltransferase is shown in red. The design of the construct is based according to Strasser et al., 2008.

```
                    *        20         *        40         *        60         *        80
044_02281  MSSNSQCLLKTLFFLLFIFPLVNARSIKSTLHKTTELDESLTYLWPLPSQFTFGNDTLTVDPNLSIVFTGNGGGSVIVKE   80
101_03794  MSSNSQCLLKTLFFLLFIFPLVNARSIKSTLHKTTELDESLTYLWPLPSQFTFGNDTLTVDPNLSIVFTGNGGGSVIVKE   80
HEXO1      MSSNSQCLLKTLFFLLFIFPLVNARSIKSTLHKTTELDESLTYLWPLPSQFTFGNDTLTVDPNLSIVFTGNGGGSVIVKE   80
AtHEXO1    MSTN---LLR--LILLFIT-----LSITSSLS-TPSPADSPPYLWPLPAEFSFGNETLSVDPTVTLIVAGNGGGSLIIRA   69

                    *       100         *       120         *       140         *       160
044_02281  AFERYKKIIFKHGSKSGDFF-----------DVTQLTVIVHSDNDELQLGVDESYSLLVTKSNEHSIIGEVSIEANSIYG  149
101_03794  AFERYKKIIFKHGSKSGDFF-----------DVTQLTVIVHSDNDELQLGVDESYSLLVTKSNEHSIIGEVSIEANSIYG  149
HEXO1      AFERYKKIIFKHGSKSGDFF-----------DVTQLTVIVHSDNDELQLGVDESYSLLVTKSNEHSIIGEVSIEANSIYG  149
AtHEXO1    AFDRYMGIIFKHASGRGSLLSRIRFLKMVEYDITSLKIVVHSDSEELQLGVDESYTLMVSKKNEQSIVGAATIEANTVYG  149

                    *       180         *       200         *       220         *       240
044_02281  ALRGLETMSQLCIFDYGVKTVQIHKAPWFIQDKPRFAYRGLLLDTSRHYLPIEIIKQIIESMSYAKLNVLHWHIIDEESF  229
101_03794  ALRGLETMSQLCIFDYGVKTVQIHKAPWFIQDKPRFAYRGLLLDTSRHYLPIEIIKQIIESMSYAKLNVLHWHIIDEESF  229
HEXO1      ALRGLETMSQLCIFDYGVKTVQIHKAPWFIQDKPRFAYRGLLLDTSRHYLPIEIIKQIIESMSYAKLNVLHWHIIDEESF  229
AtHEXO1    ALRGLETFSQLCAFDYITKSVQIYKAPWYIQDKPRFGYRGLLIDTSRHYLPIDVIKQIIESMSFAKLNVLHWHIVDEQSF  229

                    *       260         *       280         *       300         *       320
044_02281  PLEVPSYPNLWKGSYTKWERYTVEDAYEIVDFAKLRGINVMAEVDVPGHAESWGAGYPDLWPSPSCKEPLDVSKNYTFDV  309
101_03794  PLEVPSYPNLWKGSYTKWERYTVEDAYEIVDFAKLRGINVMAEVDVPGHAESWGAGYPDLWPSPSCKEPLDVSKNYTFDV  309
HEXO1      PLEVPSYPNLWKGSYTKWERYTVEDAYEIVDFAKLRGINVMAEVDVPGHAESWGAGYPDLWPSPSCKEPLDVSKNYTFDV  309
AtHEXO1    PLETPTYPNLWKGAYSRWERYTVEDASEIVRFAKMRGINVMAEVDVPGHAESWGTGYPDLWPSLSCREPLDVTKNFTFDV  309

                    *       340         *       360         *       380         *       400
044_02281  IAGILADMRKIFPFKLFHLGGDEVNTSYLLDNYATPQAMLSLFFSLQCCSYPHSVCRLQDHNMTSKDAYEYFVLRAQEIA  389
101_03794  IAGILADMRKIFPFKLFHLGGDEVNTTCWTT-------------------TPHLKQWLQDHNMTSKDAYEYFVLRAQEIA  370
HEXO1      IAGILADMRKIFPFKLFHLGGDEVNTTCWTT-------------------TPHLKQWLQDHNMTSKDAYEYFVLRAQEIA  370
AtHEXO1    ISGILADMRKIFPFELFHLGGDEVNTDCWKN-------------------TTHVKEWLQGRNFTTKDAYKYFVLRAQQIA  370

                    *       420         *       440         *       460         *       480
044_02281  ISHNWTPVNWEETFNTFPSKLNPQTVVHNWLRGGVCQAAVAKGFRCIYSNQGFWYLDHLDVPWDKVYYTEPLEGIKSISE  469
101_03794  ISHNWTPVNWEETFNTFPSKLNPQTVVHNWLRGGVCQAAVAKGFRCIYSNQGFWYLDHLDVPWDKVYYTEPLEGIKSISE  450
HEXO1      ISHNWTPVNWEETFNTFPSKLNPQTVVHNWLRGGVCQAAVAKGFRCIYSNQGFWYLDHLDVPWDKVYYTEPLEGIKSISE  450
AtHEXO1    ISKNWTPVNWEETFSSFGKDLDPRTVIQNWLVSDICQKAVAKGFRCIFSNQGYWYLDHLDVPWEEVYNTEPLNGIEDPSL  450

                    *       500         *       520         *       540         *       560
044_02281  QKLLLGGEACMWGETADASDVQQTIWPRAAAVAERLWSDKEATSSTNTTSSALQRLEYFRCLLTRRGVPAAPVTNFYARR  549
101_03794  QKLLLGGEACMWGETADASDVQQTIWPRAAAVAERLWSDKEATSSTNTTSSALQRLEYFRCLLTRRGVPAAPVTNFYARR  530
HEXO1      QKLLLGGEACMWGETADASDVQQTIWPRAAAVAERLWSDKEATSSTNTTSSALQRLEYFRCLLTRRGVPAAPVTNFYARR  530
AtHEXO1    QKLVIGGEVCMWGETADTSVVLQTIWPRAAAAAERMWSTREAVSKGNITLTALPRLHYFRCLLNNRGVPAAPVDNFYARR  530

                    *
044_02281  PPLGPGSCYEQ-  560
101_03794  PPLGPGSCYEQ-  541
HEXO1      PPLGPGSCYEQ-  541
AtHEXO1    PPLGPGSCYAQ-  541
```

Figure S2. Amino acid sequence comparison of HEXO1 sequences. The sequence alignment was done using ClustalW from the MegAlign software (DNASTAR Lasergene). The alignment was manually edited suing GeneDoc (https://www.psc.edu/index.php/user-resources/software/genedoc). 044_02281 corresponds to NbS00002281g0010.1 of the *N. benthamiana* genome v0.4.4 and 101_03794 to Niben101Scf03794g01004.1 from the current version of the *N. benthamiana* genome v1.0.1 (https://solgenomics.net/organism/Nicotiana_benthamiana/genome). The amino acid sequence of Niben101Scf03794g01004.1 is 100% identical to HEXO1. The HEXO1 ORF sequence is 100% identical to nucleotide sequences present in the *N. benthamiana* transcriptome database v5.1 (http://benthgenome.qut.edu.au/; Nbv5.1TrPrmAlt_ntDB; e.g. Nbv5.1tr6397076).

```
                    *        20         *        40         *        60         *        80
044_03864  MGKLGFRSILFAAIVQILVV---SADANIKNLKIWPMPLSVSYGHRTLLLSNDFVLKTEGSKYPDTSGILKEGFSRLLDV  77
101_02017  MGKLGFRSILFAAIVQILVV---SADANIKNLKIWPMPLSVSYGHRTLLLSNDFVLKTEGSKYPDTSGILKEGFSRLLDV  77
HEXO3      MGKLGFRSILFAAIVQILVV---SADANIKNLKIWPMPLSVSYGHRTLLLSNDFVLKTEGSKYPDTSGILKEGFSRLLDV  77
AtHEXO3    MRGSGAKIAGVLPLFMLFIAGTISAFEDIERLRIWPLPAQVSHGGRRMYLSGDFKLVTEGSKYGDASGILKEGFDRMLGV  80

                    *       100         *       120         *       140         *       160
044_03864  VKVAHVV--DANFSYAGPSPVLKGIQVVVLSPNDELQYGVDESYNLTIPAHGNPAYAYLTAKTVHGALHGLQTFSQVCHF 155
101_02017  VKVAHVV--DANFSYAGPSPVLKGIQVVVLSPNDELQYGVDESYNLTIPAHGNPAYAYLTAKTVHGALHGLQTFSQVCHF 155
HEXO3      VKVAHVV--DANFSYAGPSPVLKGIQVVVLSPNDELQYGVDESYNLTIPAHGNPAYAYLTAKTVHGALHGLQTFSQVCHF 155
AtHEXO3    VRLSHVISGDRNSSGTGGSALLQGLHVIISSSTDELEYGADESYKLVVPSPEKPSYAQLEAKSVYGALHGLQTFSQLCHF 160

                    *       180         *       200         *       220         *       240
044_03864  NFTTRTIEVHQVPWTIVDRPRFSYRGLLIDTSRHYLPLPVILKVIDSMAYAKLNVLHWHIVDTQSFPLEIPSFPKLWNGA 235
101_02017  NFTTRTIEVHQVPWTIVDRPRFSYRGLLIDTSRHYLPLPVILKVIDSMAYAKLNVLHWHIVDTQSFPLEIPSFPKLWNGA 235
HEXO3      NFTTRTIEVHQVPWTIVDRPRFSYRGLLIDTSRHYLPLPVILKVIDSMAYAKLNVLHWHIVDTQSFPLEIPSFPKLWNGA 235
AtHEXO3    NLKKKVIEILMTPWNIIDQPRFSYRGLLIDTSRHYLPLPVIKNVIDSMTYAKLNVLHWHIVDTQSFPLEIPSYPKLWNGA 240

                    *       260         *       280         *       300         *       320
044_03864  YSSSERYTVTDAAEIVRQSCRYAGRRGINVLAELDVPGHAQSWGNGYPSLWPSKDCQQPLDISNDFTFKVIDGILSDFSK 315
101_02017  YSSSERYTVTDAAEIVRQSCRYAGRRGINVLAELDVPGHAQSWGNGYPSLWPSKDCQQPLDISNDFTFKVIDGILSDFSK 315
HEXO3      YSSSERYTVTDAAEIVR----YAGRRGINVLAELDVPGHAQSWGNGYPSLWPSKDCQQPLDISNDFTFKVIDGILSDFSK 311
AtHEXO3    YSSSQRYTFEDAAEIVN----YARRRGIHVLAEIDVPGHALSWGKGYPALWPSKNCQEPLDVSSDFTFKVIDGILSDFSK 316

                    *       340         *       360         *       380         *       400
044_03864  IFKYRFVHLGGDEVDTSKL-------------------------------RQNKLNRTTAYEYFVLRAQKIALSHGYEII 364
101_02017  IFKYRFVHLGGDEVDTSKLTCSDNCPDLVVSIFLCDTLLFPWTKTLVDGLRQNKLNRTTAYEYFVLRAQKIALSHGYEII 395
HEXO3      IFKYRFVHLGGDEVDTSCWTLT---PR------------------IRKWLRQNKLNRTTAYEYFVLRAQKIALSHGYEII 370
AtHEXO3    IFKFKFVHLGGDEVNTTCWSAT---PR------------------IAQWLKKHRMSEKEAYQYFVLRAQKIALSHGYEII 375

                    *       420         *       440         *       460         *       480
044_03864  NWEETFNNFGNKLIPNTIVHNW----------------------------------------LGGGVAQQVTAAGLRCIV 404
101_02017  NWEETFNNFGNKLSRNSIVHNWCVLLSCCISWKLHISSYLFSRLFHFLYSSLPIKVIEKVRKLGGGVAQQVTAAGLRCIV 475
HEXO3      NWEETFNNFGNKLSRNSIVHNW----------------------------------------LGGGVAQQVTAAGLRCIV 410
AtHEXO3    NWEETFINFGSKLNRKTVVHNW----------------------------------------LNTGLVENVTASGLRCIV 415

                    *       500         *       520         *       540         *       560
044_03864  SNQDKWYLDHIDTTWQDFYSNEPLTNITNPKQQRLVIGGEVCMWGEHIDGSNIETTIWPRAAAAAERLWTAYDNLAKNPS 484
101_02017  SNQDKWYLDHIDTTWQDFYSNEPLTNITNPKQQRLVIGGEVCMWGEHIDGSNIETTIWPRAAAAAERLWTAYDNLAKNPS 555
HEXO3      SNQDKWYLDHIDTTWQDFYSNEPLTNITNPKQQRLVIGGEVCMWGEHIDGSNIETTIWPRAAAAAERLWTAYDNLAKNPS 490
AtHEXO3    SNQEFWYLDHIDAPWQGFYANEPFQNITDKKQQSLVLGGEVCMWGEHIDASDIEQTIWPRAAAAAERLWTPYAKLAKNPN 495

                    *       580         *       600
044_03864  QVTRRLAHFRCLLNRRGVASGPLTGGGRAAPEDPGSCYQQ- 524
101_02017  QVTRRLAHFRCLLNRRGVASGPLTGGGRAAPEDPGSCYQQ- 595
HEXO3      QVTRRLAHFRCLLNRRGVASGPLTGGGRAAPEDPGSCYQQ- 530
AtHEXO3    NVTTRLAHFRCLLNQRGVAAAPLVGGGRVVPFEPGSCLAQ- 535
```

Figure S3. Amino acid sequence comparison of HEXO3 sequences. The sequence alignment was done using ClustalW from the MegAlign software (DNASTAR Lasergene). The alignment was manually edited suing GeneDoc (https://www.psc.edu/index.php/user-resources/software/genedoc). 044_03864 corresponds to NbS00003864g0006.1 of the *N. benthamiana* genome v0.4.4 and 101_02017 to Niben101Scf02017g00001.1 from the current version of the *N. benthamiana* genome v1.0.1 (https://solgenomics.net/organism/Nicotiana_benthamiana/genome). The HEXO3 ORF sequence is 100% identical to nucleotide sequences present in the *N. benthamiana* transcriptome database v5.1 (http://benthgenome.qut.edu.au/; Nbv5.1TrPrmAlt_ntDB; e.g. Nbv5.1tr6261132).

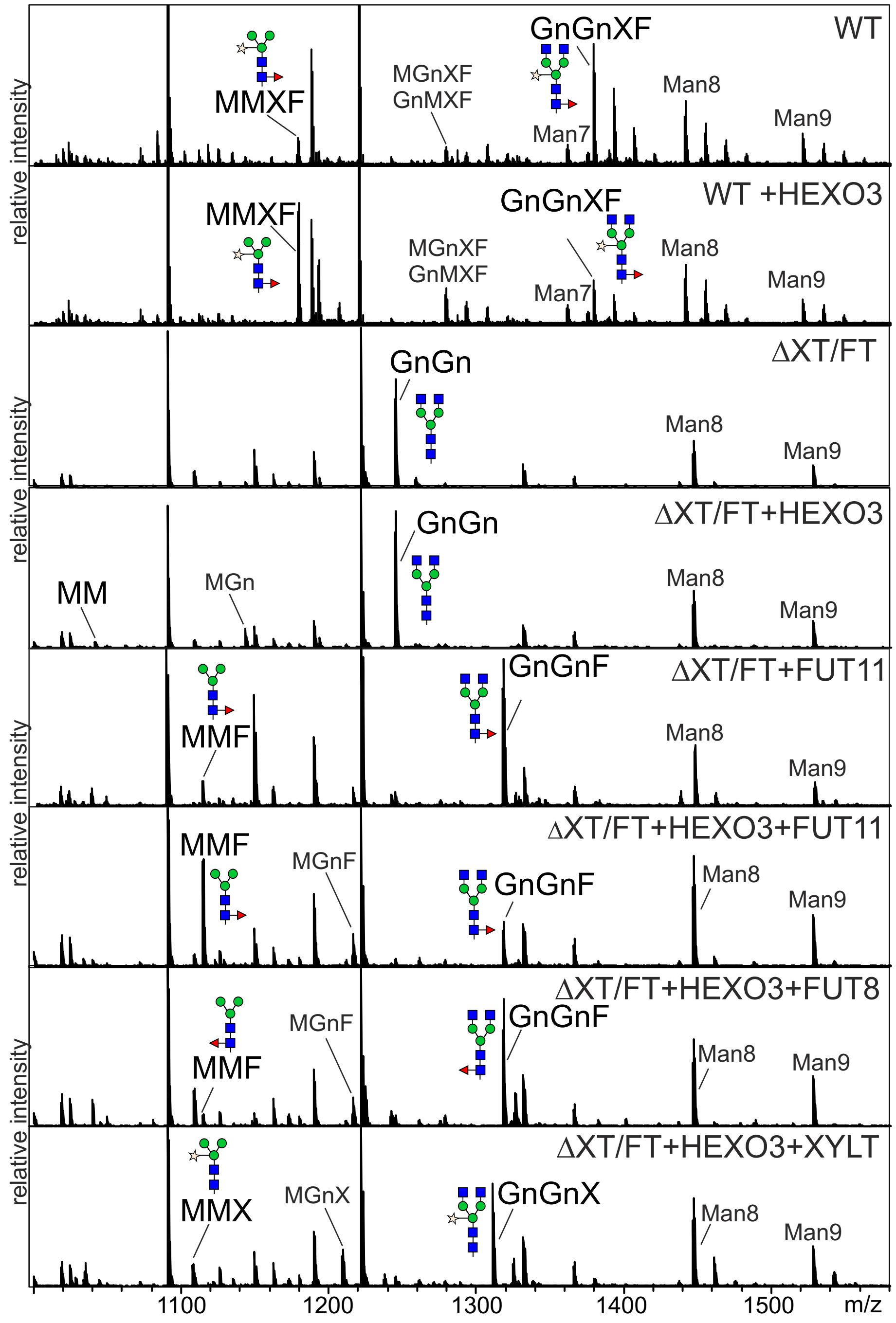

Figure S4. The presence of core α1,3-fucose enhances the trimming of GlcNAc from Fc *N*-glycans. Transient expression of Sec-Fc-mRFP and Sec-Fc-mRFP plus HEXO3-mRFP in WT or of Sec-Fc-mRFP, Sec-Fc-mRFP plus HEXO3-mRFP, Sec-Fc-mRFP plus FUT11, Sec-Fc-mRFP plus HEXO3-mRFP and FUT11, Sec-Fc-mRFP plus HEXO3-mRFP and FUT8, Sec-Fc-mRFP plus HEXO3-mRFP and XYLT in ΔXT/FT. Sec-Fc-mRFP was purified, digested with trypsin and the glycosylated peptide EEQYNSTYR was analysed by LC-ESI-MS. The spectra show $[M+2H]^{2+}$ ions.

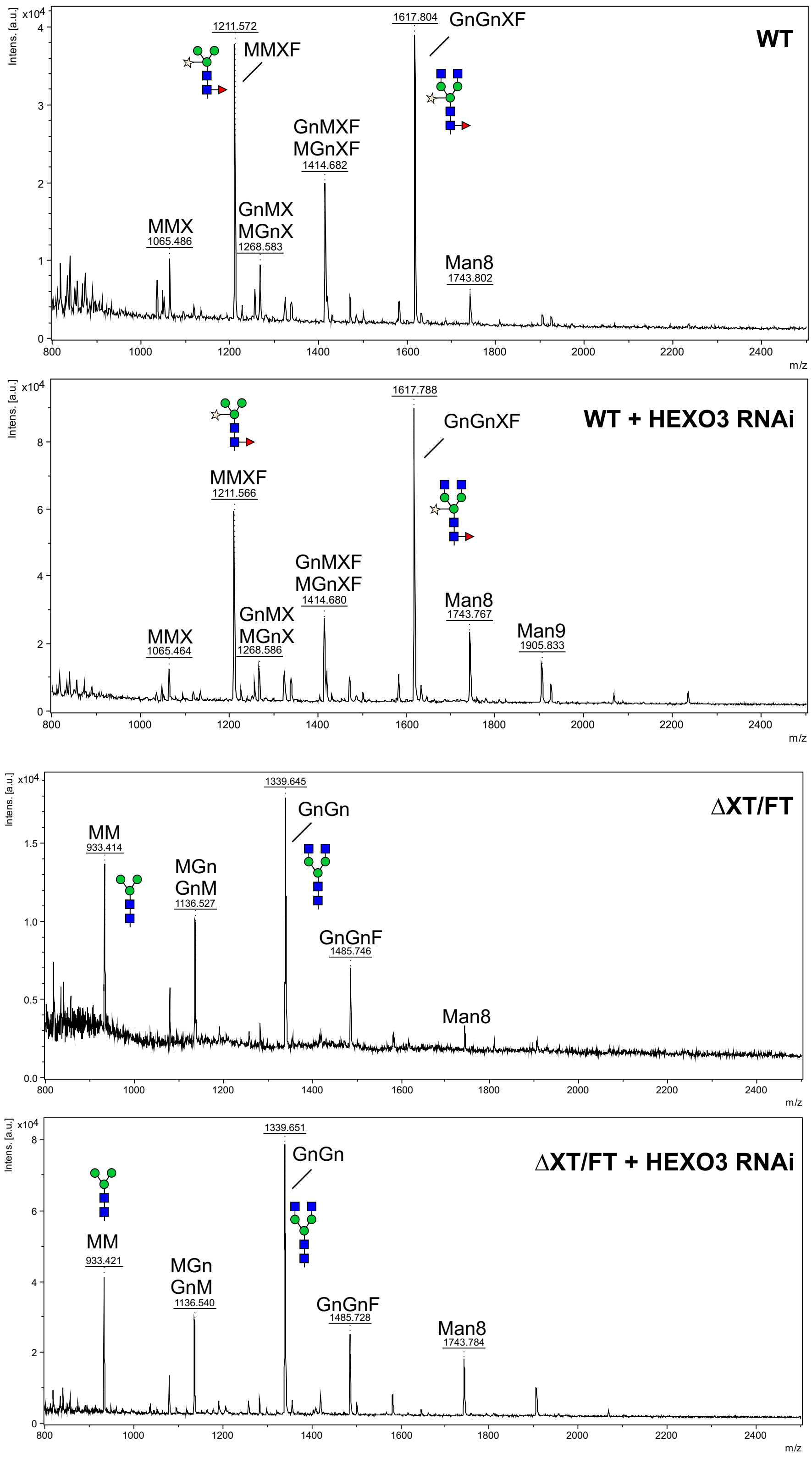

Figure S5. Effect of HEXO3-RNAi on *N*-glycans from total soluble proteins. Proteins were extracted from wild-type (WT) or ΔXT/FT leaves three days post infiltration. Total soluble proteins were digested with pepsin, the *N*-glycans released by PNGase A and analysed by MALDI-MS. The symbols for the monosaccharides in the *N*-glycan illustrations are drawn according to the nomenclature from the Consortium for Functional Glycomics (http://www.functionalglycomics.org/static/index.shtml). Peaks are labelled according to the ProGlycAn system (www.proglycan.com).

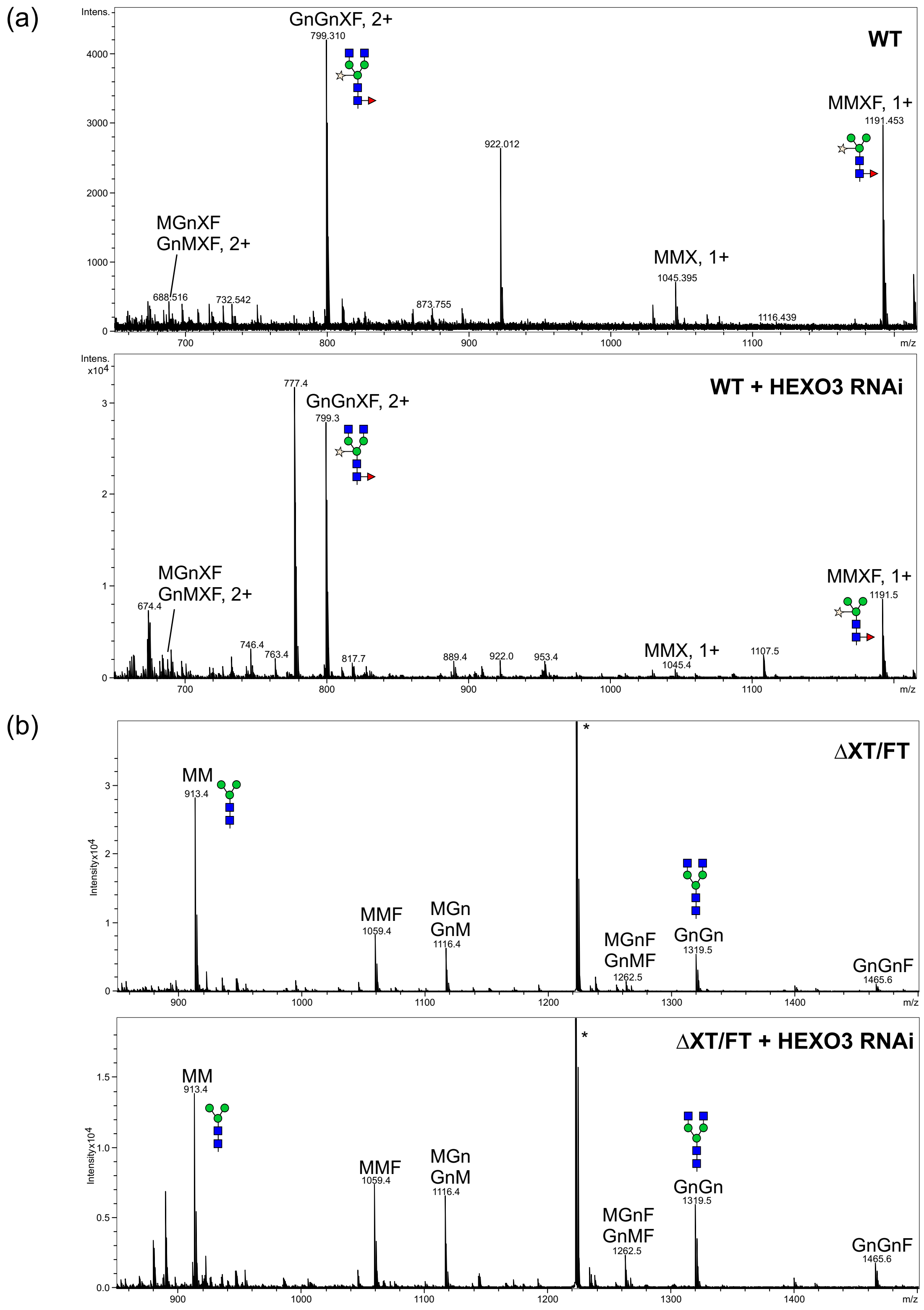

Figure S6. Effect of HEXO3-RNAi on *N*-glycans from glycoproteins of the intercellular fluid (IF). The intercellular fluid (IF) was isolated from wild-type (WT) or ΔXT/FT leaves three days post infiltration. Proteins from the IF were digested with pepsin, the *N*-glycans released by PNGase A and analysed using a Bruker ion trap system (a) or a Bruker Q-TOF mass spectrometer (b). An unspecific peak is marked with an asterisk. The symbols for the monosaccharides in the *N*-glycan illustrations are drawn according to the nomenclature from the Consortium for Functional Glycomics (http://www.functionalglycomics.org/static/index.shtml). Peaks are labelled according to the ProGlycAn system (www.proglycan.com).

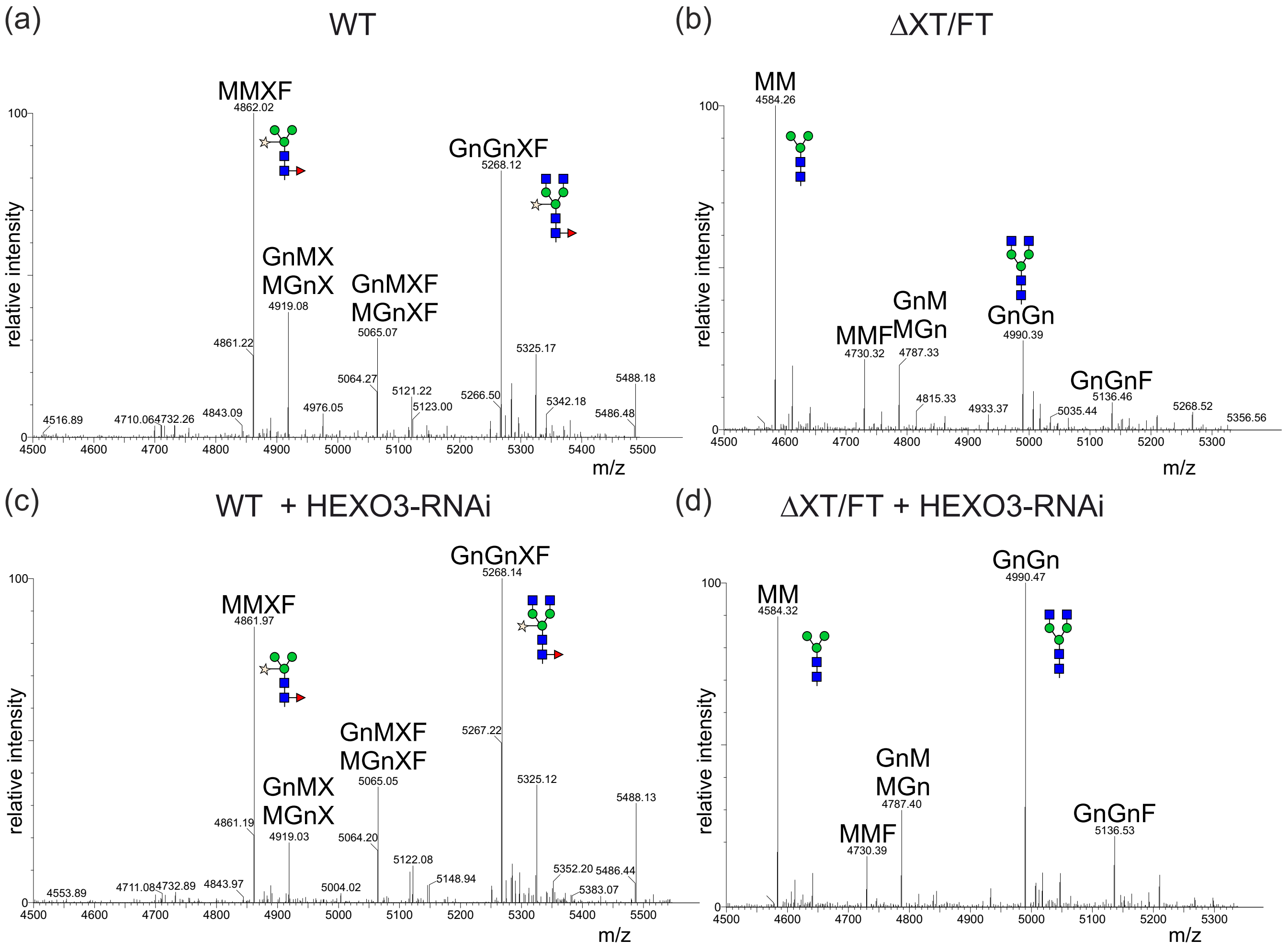

Figure S7. Transient co-expression of the HEXO3-RNAi construct leads to enhanced complex *N*-glycan formation on secreted A1AT. Human A1AT was transiently expressed in (a, c) WT or (b, d) ΔXT/FT plants, in the absence (a, b) or presence (c, d) of the HEXO3 silencing construct. LC-ESI-MS of trypsin-digested A1AT collected from the IF three days post infiltration. The *N*-glycosylation profile of glycopeptide 2 (^{70}ADTHDEILEGLNF<u>NLT</u>EIPEAQIHEGFQELLR^{101}) is shown.

Table S1. List of all primers used in this study.

For amplification of HEXO3 ORF	
Nb-Hexo3-F1	CAGTGGCTGATAATTGCATTTCCATGATAGAT
Nb-Hexo3-R1	GAATTACAACAGAGAGAGTTGCCTTACAATTTGATA
Nb-Hexo3-F2	ATTTAGTATAGTGATGGGGAAGTTAGGATT
Nb-Hexo3-R2	ACGTAACTATTGCTGATAGCAAGAACCTGGATC
For amplification of HEXO1 ORF	
Nb-Hexo1-F1	CGCCAACAGTTAAAACCAGTGACATCC
Nb-Hexo1-R1	CTATAAAGTTACAGCAAAGAGCCCGAGAA
Nb-Hexo1-F2	CCGTCTAACAAAAAATGTCCTCAAATTCCCAAT
Nb-Hexo1-R2	GCACACGGATCGAAAGATGATACAACACAAGTTA
Nb-Hexo1-F3	ACTTGGTGTTGATGAGAGCTATTCGTTATTGGTGA
Nb-Hexo1-R3	GAAGCCTGCAAACAGAATGAGGGTATGAACAGC
For cloning of HEXO1 ORF	
Nb-Hexo1-F4	TATAACTAGTATGTCCTCAAATTCCCAATGTCTT
Nb-Hexo1-R4	TATAGGATCCTTGTTCATAGCATGATCCTGGGC
For cloning of HEXO3 ORF	
Nb-Hexo3-F6	TATATCTAGAATGGGGAAGTTAGGATTCCGGAGC
Nb-Hexo3-R5	TATAGGATCCTTGCTGATAGCAAGAACCTGGATC
For cloning of the RNAi construct	
Nb-HEXO3-F8	TATATCTAGAGCAAAAACAGTTTATGGGGCGT
NB-HEXO3-R8	TATAGGTACCTCAGTTTTGCATAAGCCATTGAA
For insertion of the A. thaliana α-glucosidase II signal peptide sequence	
GCSII_SP_F	CTAGAATGAGATCTCTTCTCTTTGTACTATCACTCATTTGCTTTTGCTCTCAAACAGCACTTTCAG
GCSII_SP_R	GATCCTGAAAGTGCTGTTTGAGAGCAAAAGCAAATGAGTGATAGTACAAAGAGAAGAGATCTCATT
For cloning of A. thaliana β1,2-xylosyltransferase	
ARA_XT27F	TATAACTAGTATGAGTAAACGGAATCCGAAGATTCTG
ARA_XT29R	TATAGGATCCGCAGCCAAGGCTCTTCATGATCT
